# Does Preheating Influence the Cytotoxic Potential of Dental Resin Composites?

**DOI:** 10.3390/polym16020174

**Published:** 2024-01-07

**Authors:** Erika Katalin Dunavári, Anna Kőházy, Mónika Vecsernyés, József Szalma, Bálint Viktor Lovász, Gergely Berta, Edina Lempel

**Affiliations:** 1 Department of Restorative Dentistry and Periodontology, University of Pécs Medical School, Tüzér Street 1, 7624 Pécs, Hungary; dunavari.erika@pte.hu (E.K.D.); kohazy.anna@gmail.com (A.K.); 2 Department of Medical Biology and Central Electron Microscope Laboratory, University of Pécs Medical School, Szigeti Street 12, 7624 Pécs, Hungary; monika.hengl@aok.pte.hu (M.V.); gergely.berta@aok.pte.hu (G.B.); 3 Department of Oral and Maxillofacial Surgery, University of Pécs Medical School, Tüzér Street 1, 7624 Pécs, Hungary; szalma.jozsef@pte.hu; 4 Oral and Maxillofacial Department, Manchester University Foundation Trust, Manchester Royal Infirmary Hospital, Oxford Rd, Manchester M13 9WL, UK; balint10@hotmail.co.uk

**Keywords:** dental composite, preheated, bulk-fill, cytotoxicity

## Abstract

Resin-based dental composites (RBC) release cytotoxic components, however the extent of the elution from preheated RBCs is barely investigated. The aim was therefore to determine the cytotoxic effect of preheated conventional, bulk, and thermoviscous RBCs of clinically relevant sizes using different cell viability methods in a contact-free model. Samples (6 × 4 mm) were prepared from conventional [Estelite Sigma Quick (ESQ), Filtek Z250 (FZ)] and bulk-filled [Filtek One BulkFill Restorative (FOB), SDR Plus Bulk Flow (SDR), VisCalor Bulk (VCB)] RBCs. The pre-polymerization temperature was set to room temperature (RT) and 55/65 °C. Pulp cells were cultured, followed by a 2-day exposure to monomers released from solid RBC specimens suspended in the culture medium. Cytotoxicity was assessed using a WST-1, MTT, and LDH colorimetric viability assays. Data were analyzed using one-way ANOVA, Tukey’s post hoc test, multivariate analysis, and independent *t*-test. The effect size (ƞp^2^) of *material* and *temperature* factors was also assessed. All the RBCs demonstrated cytotoxic effect upon exposure to pulp cells, but to a varying extent (ESQ >> VCB > FZ = FOB = SDR). The effect of pre-polymerization temperature was insignificant (ƞp^2^ < 0.03), except for the thermoviscous RBC, which showed inconsistent findings when subjected to distinct viability tests. Cell viability was predominantly dependent on the type of material used (*p* < 0.001) which showed a large effect size (ƞp^2^ > 0.90). Irrespective of the pre-polymerization temperature, RBC samples in a clinically relevant size can release monomers to such an extent, which can substantially decrease the cytocompatibility.

## 1. Introduction

Resin-based dental composites (RBC) are widely used restorative materials due to their excellent esthetic and mechanical properties, handling characteristics and controlled working time [1]. To achieve a durable RBC restoration, besides the above-mentioned features, decreased polymerization stress, good marginal adaptation, and high degree of monomer to polymer conversion is desirable [2]. Although RBCs are under continuous development, they still have disadvantageous properties, depending on their type and consistency. Stickiness of conventional high-viscosity RBCs makes them difficult to handle and manipulate compromising the marginal adaptation of the restoration. Flowable RBCs demonstrate better adaptation therefore may be able to reduce marginal gaps between the cavity and the restoration, however may also undergo higher net shrinkage due to the reduced filler content, jeopardizing the physical characteristics [3,4]. Considering the advantages of high-viscosity RBCs, their viscosity can be reduced and adaptability improved by preheating [5]. In addition to the good adaptation and consequently reduced microleakage, other advantages of preheated RBCs such as increased hardness, compressive tensile strength, and a higher degree of conversion have made them popular among practitioners [6,7]. Preheated RBCs also show an increased degree of monomer to polymer conversion as a result of accelerated free radical mobility increasing the frequency at which unreactive groups and free radicals collide, which is advantageous from a biocompatibility point of view [6,8]. A higher degree of conversion (DC) reduces the solubility of the RBCs and decreased monomer elution should be expected [9]. Thermal energy transferred to the RBC system by preheating can change the polymerization kinetics, resulting in an increased DC [8]. However, rapid cooling during removal from the preheater can disturb the exothermic process of polymerization in certain types of RBCs, leading to compromised monomer-to-polymer conversion [10,11,12]. The inverse correlation found between the monomer conversion and elution raises biocompatibility issues [13]. In addition to the DC, the amount of released monomers can be influenced by, among others, the resin matrix system, the quality and quantity of the filler, the porosity of the RBC, and the type of solvent used for monomer extraction [14,15].

A recent study found distinct monomer release from preheated conventional RBCs stratified in 2 mm layers compared to 4 mm thick bulk-fill RBCs [16]. The extent of leached monomers from the tested room temperature bulk-fill RBC samples was significantly greater compared to the preheated specimens. In contrast, stratified conventional RBCs showed an increased monomer elution from the preheated samples [16]. However, it was concluded that the absolute amount of eluted resinous substances was strongly dependent on the RBC’s composition and not related to the mode of application [16]. Eluted resin monomers may have side effects such as skin, mucous membranes, and eye irritation [17]. Released monomers may penetrate to the pulp potentially inducing the production and activity of substances responsible for the mechanisms of intrapulpal monomer toxicity [18].

Regarding the cytotoxic effect of preheated RBCs, there is little and contradictory information available on this subject. By investigating the in vitro biocompatibility of preheated microfilled-hybrid RBCs, it was detected that the combination of composition, pre-polymerization temperature, curing time and polymerization pattern has a stronger effect on cytotoxicity of the RBC than the pre-polymerization temperature alone [19]. The results conducted on bulk-fill RBCs revealed no significant differences on cell viability with or without preheating, however showed the effect of RBC’s composition to be significant [20].

The aim of this in vitro study was to develop an experimental model that reproducibly enables the investigation of the cytotoxic effect of monomers released from solid RBC samples of clinically relevant sizes on cell cultures. Further aim was to determine the cytotoxic effect of conventional, bulk-fill and thermoviscous RBCs as a result of preheating using different cell viability methods. The null hypotheses were: (1) preheating of RBCs has no effect on the cell viability, and (2) there is no difference between the distinct RBCs in their effect on cell viability.

## 2. Materials and Methods

### 2.1. Reagents

All chemicals used were obtained from Millipore Sigma (Burlington, MA, USA), unless stated otherwise.

### 2.2. Pulp Cell Culturing

The study protocol was approved by the Regional Research Ethics Committee of University of Pécs (license No. PTE3026/2007). The study has been carried out in accordance with the Declaration of Helsinki principles. All data were anonymized in line with patient confidentiality guidelines. Informed consent was obtained from the subjects. Cell culture was performed as it was described by Lovász et al. [18]. Pulp tissue was isolated from caries-free third molar teeth extracted for orthodontic reasons from two individuals. Following extraction, pulp tissue was immediately isolated [21] and cultured through an explant method. For culturing minimum essential medium eagle-alpha modification (Alpha MEM) containing nucleosides, routinely supplemented with 1 mM stable Glutamine (Capricorn Scientific GmbH, Ebsdorfergrund, Germany), 10% fetal bovine serum (FBS, Euroclone, Milan, Italy), and antibiotics (100 U/mL penicillin, 100 μg/mL streptomycin, 2.5 μg/mL amphotericin B) was used. Culturing was performed at 37 °C in a humidified atmosphere containing 5% CO_2_. At 90% confluence, two or three passage to additional Petri dishes were undertaken. Cell cultures were first washed with phosphate-buffered saline (PBS, 1.37 mM NaCl, 0.27 mM KCl, 0.43 mM Na_2_HPO_4_·7H_2_O, 0.14 mM KH_2_PO_4_, pH 7.4) followed by trypsin (0.25% trypsin + 0.02% ethylene-diamine-tetraacetic acid (EDTA); Gibco, Grand Island, NY, USA) digestion for 10 min in a controlled, 37 °C environment. Cells were seeded at an arbitrary density of 2 × 10^4^ cells/cm^2^ onto 96-well plates (Greiner Bio One, Kremsmünster, Austria). Forty-eight hours prior to the start of the RBC specimen exposure, the medium was changed from 10% to 2% FBS-containing medium also free of antibiotics.

### 2.3. Resin-Based Composite Specimen Preparation

The specimens were prepared from two conventional (Estelite Sigma Quick, Filtek Z250) and three bulk-fill RBCs (Filtek One Bulk Fill Restorative, SDR Plus Flow, and VisCalor Bulk). Sample fabrication was based on a previously described method [16]. The brands, the manufacturers, the application method and pre-polymerization temperature, the acronym codes, and the chemical compositions are presented in Table 1.

The preparation of the RBC specimens was conducted under sterile conditions using sterilized instruments, devices, and single dose RBC capsules to avoid the contamination of the samples. Two experimental groups were established according to the pre-polymerization temperature of the RBC. The pre-polymerization temperature of the RBC was set at 24 ± 1 °C (room temperature—RT) in the first group, while RBCs were preheated (PH) before the sample preparation in the second group. The prewarming of the RBCs was undertaken by the Ena Heat Composite Heating Conditioner (Micerium, Avegno, Italy) using the T2 setting (55 min pre-warming of the device to 55 °C and 15 min prewarming of the RBC), except for the thermoviscous VCB, which was preheated by VisCalor Dispenser (VOCO, Cuxhaven, Germany) using the T1 setting (30 s pre-warming to 65 °C).

This preheating dispenser was specifically developed in accordance with the introduction of VCB and uses near-infrared technology for rapid warming and provides immediate application with the same device. However, this system is not compatible with the dosing capsules of other manufacturers. 

**Figure 1:**
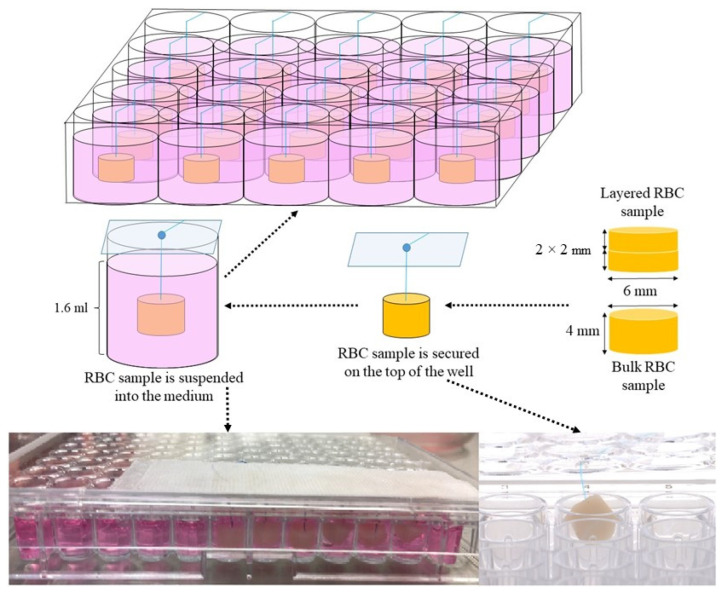
Resin-based composite (RBC) specimen preparation. The 6 × 4 mm RBC specimens were suspended in the cell culture medium using a polyethylene suture to prevent direct contact to the pulp cells.

The RBC samples were prepared in a sterilized cylindrical polytetrafluoroethylene (PTFE) mold with an internal diameter of 6 mm and a height of 4 mm, placed on a glass slide. The bulk-fill materials were used in a 4 mm-thick bulk layer; while the conventional RBCs were stratified in 2 × 2 mm layers. A sterile non-absorbable polypropylene monofilament suture thread (Prolene 6.0, Ethicon, Bridgewater, NJ, USA) was placed through the top of the RBC specimen to enable it to hang in the medium without coming into contact with the cells (Figure 1). 

Then the sample was polymerized through a polyester strip to avoid contact with oxygen. A Light Emitting Diode (LED) curing unit (LED.D, Woodpecker, Guilin, China; average light output given by the manufacturer 850–1000 mW/cm^2^; Λ = 420–480 nm; 8 mm exit diameter fiberglass light guide) in full mode for 20 s, powered by a line cord was used to irradiate the 4 mm specimens and both layers of the 2 × 2 mm samples. The light guide tip, covered with a plastic infection control barrier was centrally positioned to the mold in direct contact with the sample. The samples were irradiated only from the top to simulate clinical conditions. The irradiance of the LED unit was monitored before and after polymerization with a radiometer (CheckMARC, Bluelight Analytics, Halifax, NC, Canada). The mass of RBC specimens was measured using an analytical balance with an accuracy of 0.005 mg (Balance XPR226DRQ/M, Mettler Toledo, Greifensee, Switzerland). Volume and sample surface was also calculated.

On the sixth day of the cell culturing, the RBC specimens were suspended in the cell culture medium containing well via the secured suture. Suspending the RBC samples prevented their direct contact with the pulp cells.

### 2.4. Control Preparation

Untreated cells served as negative control. To provide positive references, pulp cells were exposed to 1.5, 3, and 6 mM triethylene glycol dimethacrylate (TEGDMA) monomer for a period of 2 days, based on literature data [22]. The different concentrations of TEGDMA monomer were prepared in the culture medium. To exclude the influencing effect of the suture used for hanging the RBC sample in the culture medium, suture incubated with cells without the RBC specimen was also tested during the viability studies.

### 2.5. Colorimetric Viability Assays

Two days after exposure to RBC samples three types of cell proliferation and viability assays were used to provide well-evidenced information about the cytotoxic effect of the compounds released from the RBCs. 

A Water-Soluble Tetrazolium Salts (WST-1) colorimetric assay, as an indicator of mitochondrial metabolism, was one of the tests employed to demonstrate changes in cell viability. 20 µL of WST-1 reagent and 180 µL of culture medium (1:9 ratio) were mixed and added to each tested well in the plate containing cultures after removing the original medium. Cells were subsequently stored at 37 °C and 5% CO_2_ for 4 h. Absorbance of 100 µL samples was measured against a background control as blank using a FluoStar Optima plate reader (BMG Labtech, Cary, NC, USA) at 450 nm.

The cell vitality was also tested through the enzymatic reduction of 3-[4,5-dimethylthiazol-2-yl]-2,5-diphenyltetrazolium bromide (MTT) to MTT-formazan, which is catalyzed by mitochondrial succinate dehydrogenase. MTT dye (Panreac Applichem ITW Reagents, Chicago, IL, USA) was dissolved in a solution of phosphate-buffered saline (PBS) at a concentration of 5 mg/mL. Then, 10 µL of the MTT solution was mixed in 100 µL of culturing medium, which was used to replace the medium on the cells inside each well. Following a 4 h incubation at a temperature of 37 °C and 5% CO_2_, the MTT containing medium on the cells was completely removed and replaced with 100 µL of an ethanol-DMSO mixture (50–50 *v*/*v*%). The optical density of the wells was determined using a FluoStar Optima plate reader against blanks at a wavelength of 595 nm.

Cellular membrane integrity was monitored using the permeability assay which is based on the release of lactate dehydrogenase (LDH) into the culture medium. The LDH-Cytotoxicity Colorimetric Assay Kit (BioVision, Milpitas, CA, USA) measures the conversion of tetrazolium salt into a formazan product. This is caused by pyruvate created from lactate through oxidation by LDH. A reaction mixture was assembled per instructions (250 µL of catalyst solution was mixed with 11.25 mL dye solution), 100 µL of which was added to 100 µL culture medium removed from the cell-containing wells. The plates were then stored at room temperature in the dark for 30 min. Subsequently absorbance was recorded at 492 nm using a FluoStar Optima plate reader using also blank reference wells. 

The data shown is a result of independent experiments carried out four times for each type of sample. The viability or cytotoxicity for each test was analyzed by normalizing absorbance values against the untreated control average of each run.

### 2.6. Statistical Analysis

Data were analyzed with SPSS version 26.0 (SPSS, Chicago, IL, USA). The Kolmogorov-Smirnov statistic was used to test the normal distribution of the data. The differences in cell viability exposed to distinct RBCs were compared with one-way analysis of variance (ANOVA), followed by Tukey’s post hoc test for multiple comparison in all ANOVA models. The relative effect size of independent factors, such as material and pre-polymerization temperature was described by general linear model and partial eta-squared (ƞp^2^) statistics. An independent two-tailed t-test was used to compare the difference between the cell viability exposed to RBCs polymerized in RT or PH form. Statistical significance was set at *p* < 0.05 for all the applied tests.

## 3. Results

The results of the three cell proliferation, viability, and cytotoxicity tests were consistent regarding the toxic effect of the investigated RT and PH RBCs. In general, both forms (RT and PH) of each RBC demonstrated a considerable toxic effect on pulpal cells. The pre-polymerization temperature of RBCs was found to have no effect on cell viability, except VCB, which showed inconsistent findings upon the application of the different test methods.

The surface of the samples was 102.1 mm^2^ and the volume was 78.5 mm^3^. The mean mass of the bulk RBC specimens was 167.3 ± 7.1 mg without significant difference (*p* = 0.42) between the RT and PH samples. The mass of layered conventional RBCs, however, showed significant difference between RT (Mean: 160.8 ± 7.4 mg) and PH (Mean: 118.8 ± 5.6 mg) specimens (*p* < 0.001).

### 3.1. WST-1 Colorimetric Viability Assay

**Figure 2:**
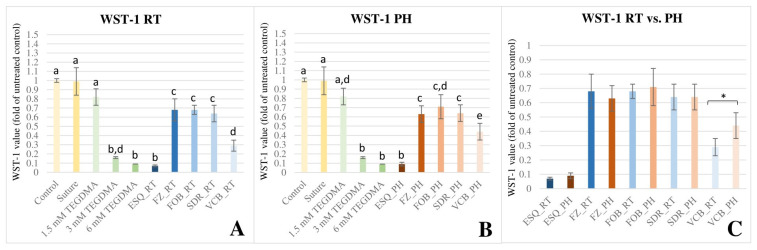
Illustration of changes in cell proliferation as detected by the water-soluble tetrazolium salt (WST-1) staining at 48 h upon exposure to the released monomers from the investigated resin-based composites (RBC). Optical densities were normed to the negative control. The graph represents the WST-1 values as a ratio compared with the average value of the untreated control. (**A**) (WST-1 RT) shows the cell viability upon exposure to RBC specimens polymerized in a room-temperature (RT) condition. (**B**) (WST-1 PH) shows the cell viability upon exposure to RBC specimens polymerized in a preheated (PH) condition. Different capital letters on (**A**,**B**) indicate a statistically significant difference according to the one-way ANOVA and Tukey’s post hoc tests. (**C**) (WST-1 RT vs. PH) illustrates the comparison of cell proliferation upon exposure to RBC specimens polymerized in RT and PH conditions. * indicates a statistically significant difference between the RT vs. PH groups according to the pairwise comparison using the independent sample *t*-test. The investigated RBCs were applied in layered [Estelite Sigma Quick (ESQ) and Filtek Z250 (FZ)] or bulk-fill [Filtek One Bulk Restorative (FOB), SDR Plus Bulk Flow (SDR), and VisCalor Bulk (VCB)] method.

According to the results of ANOVA and Tukey’s post hoc tests, WST-1 staining showed a substantially reduction in cell viability at 48 h upon exposure to each RT (Figure 2A) and PH (Figure 2B) RBCs, however, to varying extent. ESQ_RT and ESQ_PH was detected to have statistically similar (*p* > 0.05) toxic effect to the lethal doses (3 mM and 6 mM) of TEGDMA. 

The cell death observed in the case of FZ, FOB and SDR was significantly greater (*p* < 0.001) compared to the control, but similar when compared to each other (*p* > 0.05). VCB was the only RBC, where the pre-polymerization temperature played a considerable role on cell viability. According to the results of the independent t-test, significant reduction of cell viability was detected when using room-temperature VCB compared to the preheated form [t(8) = 3.06, *p* = 0.016, 95% CI = 0.03–0.27] (Figure 2C). Multivariate analysis of variance revealed that the cell viability was predominantly dependent on the type of material used (*p* < 0.001) and the effect size was considered large (ƞp^2^ = 0.90). The observed power showed the probability of correctly rejecting the null hypothesis, which assumed that there is no difference in cell viability using distinct RBCs. The power of the *material* effect was 1.000. If the study was to be replicated 100 times, the null hypothesis would be correctly rejected on 100% of those replications. However, the effect of pre-polymerization temperature was insignificant (*p* = 0.214, ƞp^2^ = 0.03) and the power of the *temperature* effect was 0.234. The effect of *material* × *temperature* interaction was also negligible (*p* = 0.134, ƞp^2^ = 0.16). 

### 3.2. MTT Colorimetric Viability Assay

**Figure 3:**
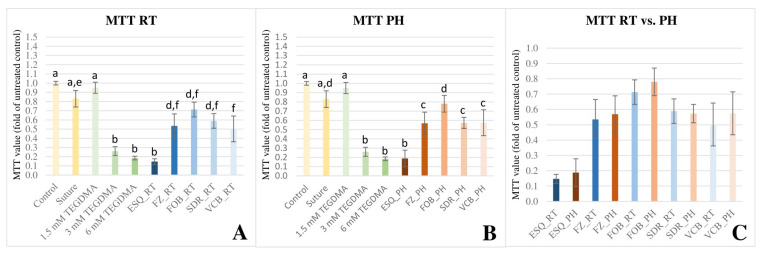
Illustration of changes in cell viability as detected by 3-(4,5-dimethylthiazol-2-yl)-2,5-diphenyl tetrazolium bromide (MTT) colorimetric assay at 48 h upon exposure to the released monomers from the investigated resin-based composites (RBC). Optical densities were normed to the negative control. The graph represents the MTT values as a ratio compared with the average value of the untreated control. (**A**) (MTT RT) shows the cell viability upon exposure to RBC specimens polymerized at room-temperature (RT). (**B**) (MTT RT) shows the cell viability upon exposure to RBC specimens polymerized in a preheated (PH) condition. Different capital letters on (**A**,**B**) indicate a statistically significant difference according to the one-way ANOVA and Tukey’s post hoc tests. (**C**) (MTT RT vs. PH) illustrates the pairwise comparison of cell viability upon exposure to RBC specimens polymerized in RT and PH conditions using the independent sample *t*-test. No statistically significant difference was found between the RT vs. PH groups. The investigated RBCs were applied in layered [Estelite Sigma Quick (ESQ) and Filtek Z250 (FZ)] or bulk-fill [Filtek One Bulk Restorative (FOB), SDR Plus Bulk Flow (SDR), and VisCalor Bulk (VCB)] method.

The results of the MTT assay confirmed the findings of the WST-1 staining as the detected changes in cell viability upon exposure to different RBCs showed a similar trend. Each investigated materials caused a considerable reduction in cell viability compared to the untreated control, irrespective of the pre-polymerization temperature applied (Figure 3A,B). In this case, the most severe toxic effect was also demonstrated upon exposure to ESQ_RT and ESQ_PH. Contrary to the results obtained from the WST-1 test, VCB—similar to the other investigated RBCs—showed no statistically significant difference in its toxic effects between the RT or PH forms using the MTT test (Figure 3C). Multivariate analysis of variance revealed that the cell viability was predominantly dependent on the type of the material used (*p* < 0.001) and the effect size was large (ƞp^2^ = 0.90). The observed power of the *material* effect was 1.000. The effect of pre-polymerization temperature was however insignificant (*p* = 0.214, ƞp^2^ = 0.03) and the null hypothesis assuming that preheating of RBCs has no effect on the cell viability would be correctly rejected on 23.4% of the replications. The effect of *material* × *temperature* interaction was also insignificant (*p* = 0.890, ƞp^2^ = 0.027). 

### 3.3. LDH Colorimetric Cytotoxicity Assay

**Figure 4:**
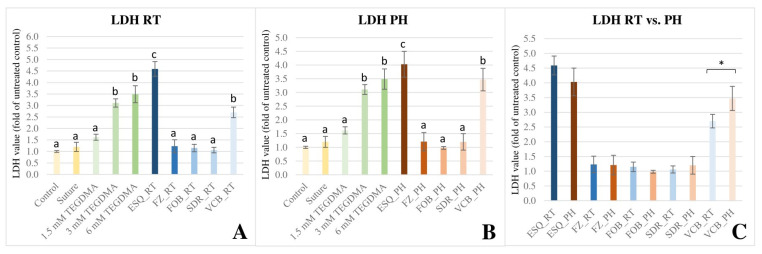
Illustration of cytotoxicity as measured by lactate dehydrogenase (LDH) assay at 48 h upon exposure to the released monomers from the investigated resin-based composites (RBC). Optical densities were normed to the negative control. The graph represents the LDH values as a ratio compared with the average value of the untreated control. (**A**) (LDH RT) shows the cytotoxicity upon exposure to RBC specimens polymerized at room-temperature (RT). (**B**) (LDH PH) shows the cytotoxicity upon exposure to RBC specimens polymerized in a preheated (PH) condition. Different capital letters on (**A**,**B**) indicate a statistically significant difference according to the one-way ANOVA and Tukey’s post hoc tests. (**C**) (LDH RT vs. PH) illustrates the comparison of cytotoxicity upon exposure to RBC specimens polymerized in RT and PH conditions. * indicates a statistically significant difference between the RT vs. PH groups according to the pairwise comparison using the independent sample *t*-test. The investigated RBCs were applied in layered [Estelite Sigma Quick (ESQ) and Filtek Z250 (FZ)] or bulk-fill [Filtek One Bulk Restorative (FOB), SDR Plus Bulk Flow (SDR), and VisCalor Bulk (VCB)] method.

The LDH assay demonstrated different results compared to the WST-1 and MTT tests. Decreased cellular membrane integrity is reflected by the increased release of lactate dehydrogenase enzyme from the pulp cells exposed to ESQ and VCB RBCs (*p* < 0.001) (Figure 4). While no differences were found comparing FZ (*p* = 0.995), FOB (*p* = 1.000), and SDR (*p* = 1.000) to the control. ESQ_RT induced a release of a large quantity of LDH that was significantly higher (*p* < 0.001) than that detected for the solution containing the absolute lethal dose of 6 mM TEGDMA. ESQ_PH (*p* = 0.154) and VCB_PH (*p* = 1.000) provided similar results to that of the 6 mM TEGDMA. By running the independent t-test, statistically significant difference [t(8) = 3.55, *p* = 0.008, 95%CI = 0.27–1.26] was only found between the VCB groups (RT vs. PH). According to this, preheating worsens the biocompatibility of the material. Similar to the previously demonstrated viability tests, multivariate analysis of variance revealed that the cytotoxicity according to the LDH assay was predominantly dependent on the type of RBC used (*p* < 0.001) and the effect size was considered to be large (ƞp^2^ = 0.95). The power of the *material* effect was 1.000. The effect of pre-polymerization temperature was insignificant (*p* = 0.995, ƞp^2^ = 0.00) and the power of the *temperature* effect was 0.05. Meanwhile, the *material* × *temperature* interaction affected the cytotoxicity significantly (*p* = 0.003, ƞp^2^ = 0.30), and the observed power was 92%.

Testing the pendant suture thread, statistically similar results with the untreated control were found with each assay, confirming that had no negative effect on the cell viability.

## 4. Discussion

Despite the gradual improvements, one of the limitations of RBCs is the incomplete polymerization [23]. The subsequent monomer release interferes with the biocompatibility of this restorative material, which otherwise has many beneficial properties and long survival rate in clinical conditions [1,18,24]. Favorable properties can be achieved not only by changing the composition, but also by the way the material is used, such as preheating before polymerization [5]. Although, improved adaptation and mechanical properties, as well as higher degree of conversion are benefits of preheating, monomer release after preheating has proven to be controversial [6,7,16]. This study aimed to investigate the cytotoxic effect of substances released from solid preheated RBC samples. The first null hypothesis, which assumed that preheating of RBCs has no effect on the cell viability was accepted, while the second, assuming that there is no difference in cell viability using distinct RBCs was rejected based on the results.

To evaluate the cytotoxic effect of the investigated RBCs both in RT and PH pre-polymerization condition, three kind of colorimetric assays targeting different aspect of cell health were applied to improve the reliability of our results. Viability as a function of cellular proliferation and death were monitored using the WST-1 and MTT assays. The cell proliferation reagent WST-1 assay is based on the cleavage of negatively charged tetrazolium salts to formazan extracellularly by mitochondrial dehydrogenases. The formation of formazan is directly proportional to the number of metabolicly active cells [25,26]. MTT is also a sensitive and reliable indicator of the cellular metabolic activity. It detects the positively charged tetrazolium salts entered intracellularly as they are reduced by oxidoreductase enzymes in viable cells [25]. LDH cytotoxicity assay is further method for determining cellular cytotoxicity which is based on the detection cytosolic enzyme (LDH) release upon damage to the plasma membrane [26]. 

The effect on cell viability and cytotoxicity of the tested RBCs coincided using the individual tests, for almost all materials. Each test revealed strong cytotoxic effect (cell viability was 5–15%) of ESQ, and slightly below the acceptable value for FOB, FZ, and SDR (cell viability was ~60–70%). VCB was the only RBC, which showed inconsistent findings using the different assays. Cell culture medium served as a negative control, meanwhile different concentrations (1.5 mM, 3 mM, and 6 mM) of TEGDMA solution were used as positive control due to its extensively studied concentration-dependent cytotoxic effect [22,27]. TEGDMA at 5 and 7.5 mM, as worst-case concentrations, inhibited proliferation and caused apoptosis, whereas no apoptosis or necrosis was observed with exposure to 1 mM or 2 mM TEGDMA [27,28]. There was a reduction in number of viable cells to 50–60% at 24 h upon exposure to 3 mM TEGDMA [22]. According to the international standard assessing biocompatibility for medical devices, a decrease in cell viability of more than 30% is considered a cytotoxic effect [29].

Our experimental model applied a non-contact exposure of cells to monomers, released from a clinically relevant size of RBC samples suspended in the culture medium. The aim of this model was to simulate a real clinical situation by exposing the cells to the same amount of monomers released from an average weight of RBC. Direct contact and mechanical load of cells were prevented by suspending the RBC specimen in the cell culture medium, allowing the examination of toxic effect directly due to the exposure to the various RBC components or derivatives. Biocompatibility testing of medical devices is recommended according to ISO 10993-12 [30,31]. This specification for eluates, providing standardized conditions and comparable results, describes that 117.8 mm^2^ sample surface area is required in 1 mL of cell culture medium. However, the ISO specification does not calculate the density or mass of the samples, although, it has been shown that the monomer release depends not only on the surface, but also on the mass of test specimen [24]. By measuring the weight of our RBC samples, it was found that preheated layered conventional RBC samples were lighter compared to the RT ones, whilst the volume and surface measurements were similar, equivalent to a moderate/large molar MOD filling. The lower viscosity of the preheated RBCs did not allow compaction of the material—which could be responsible for squeezing out air bubbles—resulting in less dense, more porous samples [32]. Undesirable porosity not only compromises the mechanical and aesthetic properties, but also strongly influences the chemical characteristics of the RBC [33]. Although, unreacted monomers are basically the result of insufficient conversion, they are also present on the inner surface of air inclusions—related to porosity formation—due to oxygen inhibition [34]. Oxygen reduces the extent of conversion by scavenging on free radicals and quenching the excited state of the initiator [35]. The DC can be as low as 25–35% if oxygen is in contact with the resin [36]. A previous study evaluating the relation of porosity and monomer elution demonstrated, that although preheating increased the porosity of both conventional and bulk-fill RBCs, the monomer release was significantly elevated only in the case of layered RBCs [16]. However, our results regarding the cytotoxicity of preheated RBCs did not reflect the aforementioned differences. Based on the unanimous findings of all three cytotoxicity tests, the general linear model and partial eta-squared statistics revealed an insignificant effect of preheating. Similar to our results, negligible effect of preheating was observed in other studies also, in contrast to the effect of the type of RBCs which resulted in expressed differences in cell viability [20,37]. Undesirable effects are attributed to monomers, which are a significant component of RBCs as they represent about 20–40% of their content. The 60–80% inorganic filler content does not seem to play a major role in the biocompatibility of these materials. Additives, such as polymerization promoters, modifiers or inhibitors represent only 1–3% of the composition [38]. The order of monomers in terms of their cytotoxic potential is BisGMA > UDMA > TEGDMA > HEMA [39]. Cell viability reduction of 50% was reported after exposure of human gingival fibroblasts to BisGMA at a concentration of 0.087 mmol/L, to UDMA at 0.106 mmol/L, to TEGDMA at 3.460 mmol/L, and to HEMA at 11.530 mmol/L [39]. The reduction of cell viability was related to the increased amount of reactive oxygen species, oxidative stress, cell cycle disruption, DNA strand damage and signaling alterations among others [18,40,41]. However, not only individual monomers, but the combinations of them also have a variable effect on the cells. A greater cytotoxic and genotoxic potential of RBC was observed in the case of the joint release of BisGMA and TEGDMA [42]. Regarding the material’s type, ESQ supra-nanofilled conventional layered RBC—which is a TEGDMA- and BisGMA-based RBC—showed the worst results in terms of cytotoxicity in both RT and PH groups. A recent study with the same sample geometry demonstrated ~ 3.5–4 nmol (RT) and 4–5 nmol (PH) TEGDMA and BisGMA monomer release from 1 mg RBC sample using a 75% of ethanol-water solvent [16]. Considering the hereby measured weight of the RT (~160 mg) and PH (~120 mg) samples, ESQ can elute roughly 600 nmol TEGDMA and BisGMA irrespective of their pre-polymerization temperature which was calculated with the above referred data. Although, statistically significant difference was found in monomer elution between RT and PH samples based on the above-mentioned study [16], the difference in mass of ESQ specimens offsets the amount of released monomers, ensuring a similar toxicity in the RT and PH groups. This relationship is true also for the other tested RT and PH RBCs. Moreover, in the present study a lower monomer release was expected than the above calculation, since the cell culture medium was water-based in contrast to the cited article where a 75% ethanol-based extraction medium was employed. Elution into water-based solvents was found to be lower, although the dynamics of dissolution is significantly influenced by the nature of the monomer [24,43]. Furthermore, in the referenced elution study [16] the RBC specimens were soaked for 72 h, meanwhile, the detection of cytotoxicity in the present investigation occurred after 24 h, with the expectation of less components released from the samples. Parallel to the maximum degree of monomer to polymer conversion, however, it has been reported that the maximum release of unreacted monomers occurs in the first 24 h after polymerization [44,45]. In addition to the resin-matrix components, highly filled (83 w%) ESQ contains TiO_2_ nanoparticles, which were reported to induce cell apoptosis and necrosis, furthermore increase reactive oxygen species concentration [46]. On the other hand however, it was stated that high filler proportion resulted in higher cell viability, which can be attributed to the presence of a smaller proportion of soluble resin [47]. The advantage of the high filler content regarding cell viability cannot be obviously supported by the results of our study.

FZ conventional layered RBC also contains BisGMA and TEGDMA, as does ESQ, but in addition BisEMA and UDMA form the basis of the resin matrix. It is assumed, that copolymerization of its monomers results in synergistic effects on double bond conversion and rotational freedom, thus increasing polymer network homogeneity [48]. Similar to our findings, it was concluded in a research testing the cytotoxic effect of highly esthetic RBCs, that light-cured particulate filled RBCs containing BisGMA, TEGDMA, UDMA and BisEMA monomers in their organic matrix show acceptable cell viability as specified by the International Standards Organization (ISO) (70%) [49]. This was also indirectly proven by the very low rate of monomer dissolution both from FZ_RT and FZ_PH samples [16]. A previous study determined that all of the different components simultaneously present in the RBC may induce effects that are different from what would be expected by their action alone, as they can mutually influence each other through synergistic and/or antagonistic behaviors [50]. 

FOB and SDR bulk-fill RBCs in the present study showed also lower level of toxic effect similar to FZ. The toxic effect of these RBCs was equivalent to that of TEGDMA between 1.5 and 3 mM, which reflects the low amount of eluted monomers detected by previous studies [14,16]. FOB and SDR are UDMA-based materials with other dimethacrylates. In addition, FOB contains a so-called addition fragmentation monomer (AFM), which includes a complementary internal double bond with a β-quaternary carbon center functional group [51]. It is assumed that the living polymerization resulting from AFM incorporation is responsible for the increased DC, even in a 4-mm-thick bulk increment, leaving fewer monomers unreacted [51]. Due to the presence of a photo-active modulator in SDR’s matrix system and the increased translucency, higher DC and lower monomer elution could be responsible for the lower cytotoxic effect compared to the ESQ. 

The chemical composition of RBC has a great impact on the degree of monomer to polymer conversion [52]. Although the DC of RBC’s was not measured in this study—which can even be mentioned as one of the limitations of this experiment –, it is supposed, that the differences in the degree of polymerization are primarily responsible for the observed differences in toxicity. However, despite the inverse relationship between the DC and monomer elution [53], it was demonstrated that lower DC of RBCs do not necessarily lead to distinct cytotoxic effects [54]. Apart from RBC composition, however, the cell viability tested in another study was influenced by a different combination of curing time, pre-polymerization temperature, and polymerization pattern [19]. Previous studies have reported controversial effect of the pre-polymerization temperature on the degree of conversion. On one hand, the preheating of RBC can enhance the rate of polymerization and the degree of conversion by promotion of reactive radical mobility [8]. On the other hand, rapid cooling during removal from the heating device may compromise the monomer-to-polymer conversion [2,55]. The application method was found to have a great impact on the monomer release in relation to the rapid cooling [16]. While the evaluated monomers from the bulk-fill RBCs showed a significantly greater degree of elution when applied at RT, the elution of monomers increased from layered conventional RBCs, when their pre-polymerization temperature was elevated [16]. These results were not reflected by the toxicity tests, only VCB thermoviscous bulk-fill RBC presented contradictory results using the distinct viability tests. WST-1 test showed a higher toxic effect of VCB_RT, while LDH test revealed the same for VCB_PH. The results of MTT test showed consistent findings with the other RBCs, without significant difference between RT and PH samples. It was demonstrated, that despite the reduced monomer elution seen with the application of preheating, the absolute amount of BisGMA was the highest from VCB (8.6 nmol/1 mg RT RBC and 6.6 nmol/1 mg PH RBC) when compared to the other investigated BisGMA-based bulk-fill RBCs [16]. Increasing concentrations of BisGMA can cause cell death varying from apoptosis to necrosis [56]. A key sign of necrosis, as a result of massive cell damage, is the permeabilization of cell membrane leading to leakage of organelles [57]. In the case of VCB_PH, this is suggested from the increased LDH release, as in the case of ESQ_RT and ESQ_PH. Reflecting on the disparate result of the WST-1 assay, it is speculated, that the extremely rapid cooling (~35–36 °C) during the application of the VCB due to heat equalization between the preheated material and the ambient temperature [55,58,59] may hinder the exothermic temperature increase, thereby disrupting and destabilizing the polymerization kinetics, leading to heterogeneous network and inconsistent unbonded components [10].

Overall, these results confirm an extended, composition-dependent cytotoxic potential of RBCs. Since these findings were observed on a cell culture system, they cannot be directly extrapolated to in vivo situations. However, these results highlighted the need for designing future studies aiming to further clarify or prevent mechanisms involved in the cytotoxic action of daily used RBCs or their preheated counterparts. Researches on the relationship between polymerization kinetics and dissolved monomer combinations and cytotoxicity are also necessary. For this purpose, our in vitro model system allows the deeper analysis of the immersed RBCs’ isolated and precise effect in a controlled system free of environmental and disturbing factors also associated with living organisms. 

Although the tests were performed on pulp cells, the model simulated the effect on the oral mucosa cells. To reproduce a real clinical scenario, a delimiting layer of dentin should be placed between the RBC and the culture medium allowing penetration of the released components through the dentinal tubules. The lack of this is a limitation of our study. 

## 5. Conclusions

Within the limitations of this in vitro study the following conclusions can be drawn regarding the influence of RBC preheating on cell viability:

- Preheating had no significant contributing effect based on the low value of effect size (ƞp^2^ > 0.03).
- The exposure to components released from conventional layered, bulk-fill and thermoviscous RBCs resulted in significant negative biological response from pulpal cells, which indicates a large effect size (ƞp^2^ > 0.90) of the material used.
- Each cell viability assay revealed composition-dependent strong cytotoxic effect and the cell viability exceeded the 30% limit value considered accepted for all tested RBCs.

## Figures and Tables

**Table 1:** Composition, manufacturer, pre-polymerization temperature (Temp), and application method (Appl. mode) of the tested resin-based composites (RBC).

RBC (LOT Number)	Manufacturer	Temp	Appl. Mode	Group Code	Matrix	Fillers	Filler-Load
Estelite Sigma Quick E8726	Tokuyama, Tokio, Japan	24 °C	Layered (2 × 2 mm)	ESQ_RT	TEGDMA, BisGMA	0.2 µm spherical Si-Zr, TiO_2_	71 vol% 82 wt%
		55 °C		ESQ_PH			
Filtek Z250 9438725	3M ESPE, St. Paul, MN, USA	24 °C	Layered (2 × 2 mm)	FZ_RT	BisGMA, BisEMA, TEGDMA, UDMA	0.01–3.5 µm (mean 0.6 µm) Zr-silica	60 vol% 80 wt%
		55 °C		FZ_PH			
Filtek One Bulk Fill Restorative 40213	3M ESPE, St. Paul, MN, USA	24 °C	Bulk (4 mm)	FOB_RT	AFM, UDMA, AUDMA, DDDMA	20 nm silica, 4–11 nm Zr, Zr-silica groups, 0.1 µm YbF_3_	58.5 vol% 76.5 wt%
		55 °C		FOB_PH			
SDR Plus Bulk Flow	Dentsply, Milford, DE, USA	24 °C	Bulk (4 mm)	SDR_RT	Modified UDMA, TEGDMA, di-, trimethacrylates	Ba-Al-F-B-silicate glass, Sr-Al-F-silicate, YbF_3_	47.4 vol% 70.5 wt%
		55 °C		SDR_PH			
VisCalor Bulk 2127287	Voco, Cuxhaven, Germany	24 °C	Bulk (4 mm)	VCB_RT	BisGMA, aliphatic dimethacrylate	Inorganic nano-hybrid fillers *	83 wt%
		65 °C		VCB_PH			

Abbreviations: RT: room-temperature; PH: preheated; BisGMA: bisphenol-A diglycidyl ether dimethacrylate; AFM: addition fragmentation monomer; UDMA: urethane dimethacrylate; AUDMA: aromatic urethane dimethacrylate; DDDMA: 1,12-dodecane dimethacrylate; TEGDMA: triethylene glycol dimethacrylate; vol%: volume%; wt%: weigth%; * did not specified by the manufacturer.

## Data Availability

The datasets generated during and/or analyzed during the current study are available from the corresponding author on reasonable request.
